# Maintenance and Representation of Mind Wandering during Resting-State fMRI

**DOI:** 10.1038/srep40722

**Published:** 2017-01-12

**Authors:** Ying-hui Chou, Mark Sundman, Heather E. Whitson, Pooja Gaur, Mei-Lan Chu, Carol P. Weingarten, David J. Madden, Lihong Wang, Imke Kirste, Marc Joliot, Michele T. Diaz, Yi-Ju Li, Allen W. Song, Nan-kuei Chen

**Affiliations:** 1 Department of Psychology, University of Arizona, Tucson, AZ, USA; 2 Cognitive Science Program, University of Arizona, Tucson, AZ, USA; 3 Arizona Center on Aging, University of Arizona, Tucson, AZ, USA; 4 Department of Medicine and Ophthalmology, Duke University Medical Center, Durham, NC, USA; 5 Geriatrics Research Education and Clinical Center, Durham Veterans Administration Hospital, Durham, NC, USA; 6 Institute of Imaging Science, Vanderbilt University, Nashville, TN, USA; 7 Brain Imaging and Analysis Center, Duke University Medical Center, Durham, NC, USA; 8 Department of Psychiatry and Behavioral Sciences, Duke University Medical Center, Durham, NC, USA; 9 Department of Psychiatry, University of Connecticut Health Center, Farmington, CT, USA; 10 Neuroimaging Group (GIN), UMR5293, CEA CNRS Université de Bordeaux, Bordeaux, CEDEX, France; 11 Department of Psychology, Penn State University, University Park, PA, USA; 12 Department of Biostatistics and Bioinformatics, Duke University Medical Center, Durham, NC, USA; 13 Department of Radiology, Duke University Medical Center, Durham, NC, USA; 14 Department of Biomedical Engineering, University of Arizona, Tucson, AZ, USA; 15 Department of Medical Imaging, University of Arizona, Tucson, AZ, USA

## Abstract

Major advances in resting-state functional magnetic resonance imaging (fMRI) techniques in the last two decades have provided a tool to better understand the functional organization of the brain both in health and illness. Despite such developments, characterizing regulation and cerebral representation of mind wandering, which occurs unavoidably during resting-state fMRI scans and may induce variability of the acquired data, remains a work in progress. Here, we demonstrate that a decrease or decoupling in functional connectivity involving the caudate nucleus, insula, medial prefrontal cortex and other domain-specific regions was associated with more sustained mind wandering in particular thought domains during resting-state fMRI. Importantly, our findings suggest that temporal and between-subject variations in functional connectivity of above-mentioned regions might be linked with the continuity of mind wandering. Our study not only provides a preliminary framework for characterizing the maintenance and cerebral representation of different types of mind wandering, but also highlights the importance of taking mind wandering into consideration when studying brain organization with resting-state fMRI in the future.

Over the past two decades, resting-state functional connectivity measured by functional magnetic resonance imaging (fMRI) has played an essential role in understanding brain functional networks in healthy and patient populations^1^,^2^,^3^,^4^,^5^. Resting-state functional connectivity is measured by the temporal co-activation level of spontaneous fMRI signals between spatially distinct brain regions in the absence of a perceptual or behavioral task^6^. Although the participants are not engaged in any particular task, there is increasing evidence that spontaneous thoughts (known as mind wandering, daydreaming, self-generated mental activity or task-unrelated thought) that are minimally constrained by external perception emerge during fMRI scans and may potentially affect resting-state fMRI data^7^,^8^. Mind wandering during resting-state fMRI has been assessed using different approaches. Questionnaires can be administered, following the resting-state fMRI scan, in which participants are asked to report the presence and frequency of spontaneous thoughts across various domains. Resting-state fMRI studies have employed several types of retrospective measures to assess spontaneous thoughts: Amsterdam Resting-State Questionnaire (ARSQ)^9^, New York Cognition Questionnaire (NYC-Q)^10^,^11^,^12^, and Resting-State Questionnaire (ReSQ)^13^,^14^. Alternatively, mind-wandering has been assessed using experience or thought sampling in conjunction with resting-state fMRI scanning^15^,^16^,^17^,^18^,^19^,^20^. While regions within the default mode network are involved in mind-wandering, a number of other brain regions outside the default mode network also show associations with various contents and forms of spontaneous thoughts^10^,^15^,^16^,^21^,^22^. These findings contribute to an increasingly diverse and complex understanding of the spontaneous thoughts that may occur during resting-state fMRI scans, and thus provoke more questions on the impact of mind-wandering on fMRI data.

For example, previous studies using the ReSQ have indicated that, on average, participants reported spending about 40% and 30% of time on visual and auditory mental imagery, respectively, during resting-state fMRI scans^5^,^13^,^14^. The remaining portion of the scan was filled with a variety of spontaneous thought domains including those pertaining to somatosensory awareness, inner musical experience, and manipulation of numbers^13^,^14^. This gives rise to the questions that form the analytical focus of our study. How is the continuity of spontaneous thoughts supported? Is the mechanism underlying the support of spontaneous thoughts comparable across different domains? Are different thought domains represented by divergent functional connections across the cerebral cortex? Recent studies have observed the non-static nature of resting-state functional connectivity across a single fMRI scan^23^,^24^,^25^,^26^,^27^. Will regulation of mind wandering contribute to the temporal changes in resting-state functional connectivity?

To address these questions, first, we employed multiple regression analyses to identify functional connections that exhibited a significant group difference in connectivity between participants who spent more time in a self-reported spontaneous thought and participants who spent less time in the same thought domain during resting-state fMRI (e.g., those who reported spending a lot of time in auditory mental imagery compared to those who reported spending little or no time on such wandering thought). The functional connections exhibiting a significant group difference in connectivity for a specific spontaneous thought domain would be indicative of the neural correlates associated with sustaining this spontaneous thought. Second, we investigated whether group effects on functional connectivity would vary between earlier and later parts of the resting-state fMRI data time points. Our goal is to provide a framework for studying the maintenance and cerebral representation of mind wandering, and understanding the impact of mind wandering on the acquired resting-state fMRI data.

## Results

### Behavioral Responses

**Figure 1:**
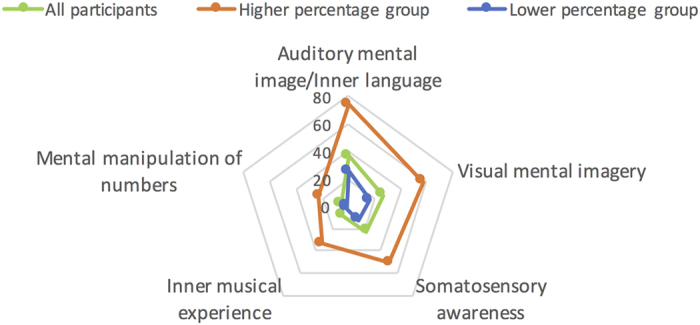
Estimated proportion of time spent in each thought domain of mind wandering.

Each participant completed a post-resting-state-fMRI interview using the Resting-State Questionnaire (ReSQ)^13^ to assess spontaneous thoughts during the resting-state fMRI scans. Participants were asked to estimate the proportion of time (on a 0–100% scale) spent during the resting-state fMRI scans in each of the following five spontaneous thought domains: auditory mental imagery/inner language (AUDI/LANG), visual mental imagery (VIMG), somatosensory awareness (SEN), inner musical experience (MUS), and mental manipulation of numbers (NUM). Descriptions of each thought domain are included in the Methods section. On average, the participants reported spending the greatest amount of time in the AUDI/LANG (36.7%) domain, followed by VIMG (26.1%), SEN (22.5%), MUS (8.5%), and NUM (6.2%). For data analyses of each domain of spontaneous thought, participants were split into two groups (higher vs. lower percentage groups). The higher percentage group included participants whose estimated percentage of time spent in a specific thought domain was greater than the 75^th^ percentile (i.e., upper quartile across all the participants), while the lower percentage group included the remainder of the participants. No significant differences in age were found between the two groups, for any of the individual thought domains. Figure 1 illustrates the estimated proportion of time spent in each thought domain for the higher and the lower percentage groups.

### Matrix-Based Connectivity Analysis Results

**Figure 2:**
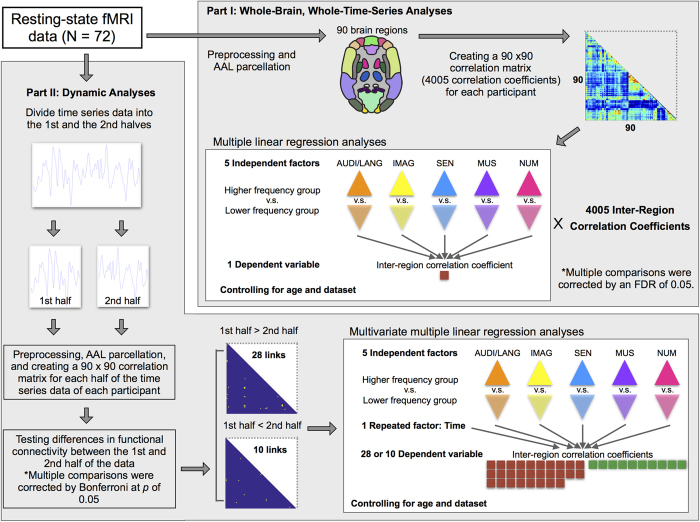
Summary of functional connectivity analysis procedures.

In contrast to seed-based analysis that relies on prior knowledge for choosing seed regions, the matrix-based approach employed in this study thoroughly examines functional connectivity between every pair of regions across the whole brain. Our matrix-based functional connectivity analysis procedures are described in detail in the Methods section and illustrated in Fig. 2.

#### Part I: Whole-brain, whole-time-series analyses

The first goal of our study was to identify functional connections that exhibited significantly different connectivity between groups of higher- and lower-frequency of mind wandering during fMRI scans (see Part I in Fig. 2). The resting-state fMRI data were preprocessed and parceled into a set of 90 brain regions using Automated Anatomical Labeling (AAL) template^28^. Inter-regional functional connectivity was estimated using the pairwise Pearson correlation statistics, resulting in 4005 ([90 × 89]/2) correlation coefficients for each participant. We examined group effects on functional connectivity of individual links by performing multiple linear regression analysis 4005 times. Each regression model included 5 independent factors (i.e., group effects of each thought domain), with each controlled for the others, and 1 dependent variable (i.e., the functional connectivity value of an individual link). Our resting-state data were aggregated from three unpublished datasets (see Methods section). Therefore, we added the “dataset” as a covariate in the regression models to control for any variability across datasets.

**Figure 3:**
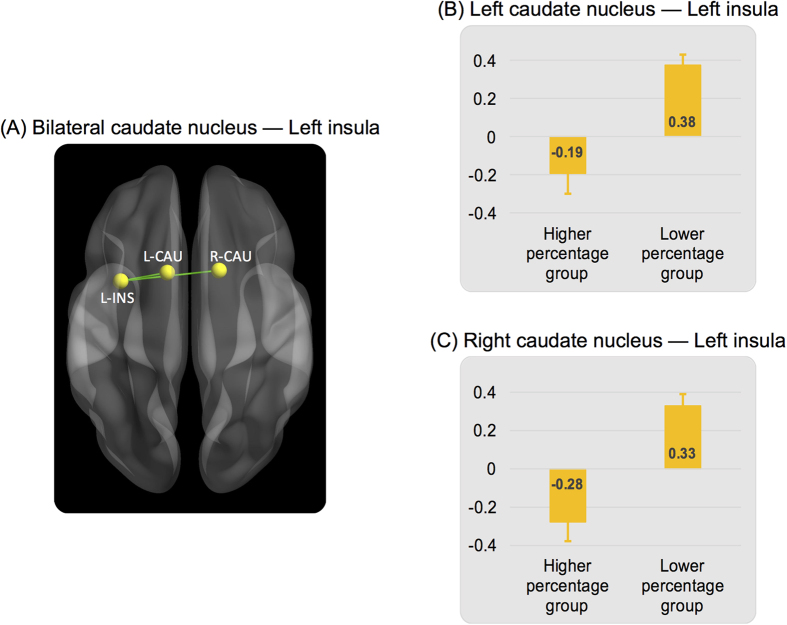
(**A**) Functional connections between the left insula (L-INS) and bilateral caudate nuclei (CAU) were associated with the continuity of spontaneous thought for auditory mental imagery/inner language (AUDI/LANG). Data were derived from whole time series data. (**B**) and (**C**) Participants who reported spending more time in mind wandering associated with AUDI/LANG (higher percentage group) exhibited a more negative functional connectivity compared to participants who reported spending less time in AUDI/LANG (lower percentage group). Error bars denote standard errors.

The analyses yielded 2 significant functional links for the thought domain of AUDI/LANG, corrected for multiple comparisons at a false discovery rate (FDR) of 0.05^29^. The two functional links (Fig. 3A) were connected between the left insula and the left caudate nucleus, *t*(65) = −4.64, *p* = 0.000017, and between the left insula and the right caudate nucleus, *t*(65) = −5.01, *p* = 0.000004. For both functional links, participants in the higher percentage group for AUDI/LANG exhibited a significantly more negative connectivity relative to the participants in the lower percentage group (Fig. 3B and C). As described in the Discussion section, bilateral caudate nuclei are brain regions involved in brain state maintenance, and the left insula supports switching between different mental states^30^. No significant associations with functional links were identified for other thought domains. The results suggest that the decrease in functional connectivity of connections between the left insula and bilateral caudate nuclei was associated with the continuity of spontaneous thought related to AUDI/LANG.

#### Part II: Dynamic analyses

The second goal of our study was to investigate whether group effects on functional connectivity links would vary between earlier and later portions of the resting-state time series data (see Part II in Fig. 2). To this end, we examined whether there was an interaction effect between group and timing of the resting-state fMRI data time course profiles. First, we divided each participant’s time series data into halves (i.e., the 1^st^ half and the 2^nd^ half of the time series data, see Fig. 2). For each half, the preprocessed fMRI data were parceled using the AAL template^28^, and 4005 ([90 × 89]/2) correlation coefficients were estimated for each half of the time series data of each participant (as stated in the previous section). We then examined differences in functional connectivity between the 1^st^ half and the 2^nd^ half of the time series data by performing paired sample *t* tests on each connectivity value of the 4005 inter-regional functional links.

**Figure 4:**
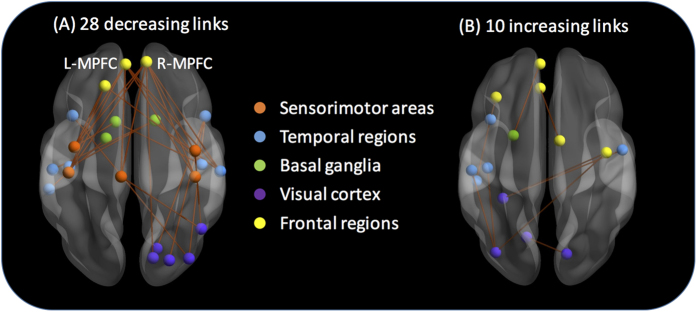
Functional links that exhibited a significant difference in connectivity between the 1^st^ and the 2^nd^ halves of the fMRI time series data. (**A**) The majority of the decreasing links were connected to the bilateral medial prefrontal cortex (MPFC), primary sensorimotor cortex, and temporal regions. (**B**) The increasing links were distributed among visual, temporal, and frontal areas.

The analysis yielded 38 functional links for which mean connectivity differed significantly between the first and second halves of the scan, with Bonferroni correction for multiple comparisons (alpha = 0.05/4005 ≈ 0.000012) to minimize false positives. Among the 38 links, 28 links exhibited decreased functional connectivity from the 1^st^ half to the 2^nd^ half of the time series data. These links temporally changed their connectivity either from positive to negative, from more positive to less positive, or from less negative to more negative and we called these 28 links “decreasing links”. An additional 10 links exhibited increased temporal functional connectivity in the second half of the scan and they are termed “increasing links”. Among the decreasing links (Fig. 4A), most links were connected to the bilateral medial prefrontal cortex (MPFC), primary sensorimotor area, and temporal regions. For the increasing links (Fig. 4B), connections were dispersed among visual, temporal, and frontal areas. Additional details of the decreasing and increasing links are presented in Supplementary Table S1. We then converted the resultant 2 sets of links (i.e., decreasing and increasing links) into 2 binary matrices and used them as inclusive masks in the subsequent analysis.

Within each resultant set of links, we examined Group × Time interaction effects on functional connectivity using a multivariate multiple regression analysis, which estimated a single regression model with more than one dependent variable. The main advantage of the multivariate multiple regression analysis is that all the assessments can be performed in a single step, and thus the risk of false positives associated with repeated assessments (i.e., multiple comparisons) in conventional univariate multiple regression can be inherently eliminated. This analysis was chosen to account for the relationships among several dependent variables and conduct tests of the coefficients across different variables. Our model included 5 independent factors (i.e., group effects of each thought domain), 1 repeated factor (i.e., time: 1^st^ half vs. 2^nd^ half of the time series data), 1 covariate (i.e., dataset), and functional connectivity values for a set of links (either all decreasing links or all increasing links) as dependent variables. The multivariate multiple regression analysis yielded two outputs: 1) results of multivariate analysis of variance that tested the overall group effects on functional connectivity across all dependent variables; and 2) results of univariate analysis that examined the group effect on the functional connectivity of each individual dependent variable for each thought domain.

**Figure 5:**
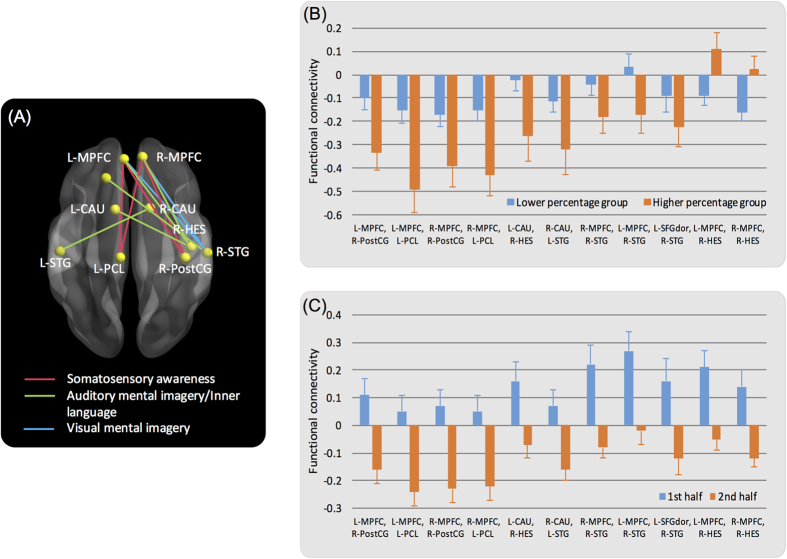
(**A**) Eleven functional connections were associated with the continuity of spontaneous thoughts for somatosensory awareness (red), auditory mental imagery/inner language (green), and visual mental imagery (blue). These connections were identified from the 2^nd^ half time series data of the decreasing links. Spheres represent the centroids of the Automated Anatomical Labeling Template regions as estimated by the BrainNet Viewer^70^. (**B**) Participants in the higher percentage group (orange) exhibited more negative functional connectivity in the majority of links relative to the lower percentage group (blue). Functional connectivity was estimated from the 2^nd^ half of the time series data. (**C**) Functional connectivity significantly decreased from the 1^st^ half (blue) to the 2^nd^ half (orange) of the time series data. Error bars denote standard errors. Abbreviations: L = left; R = right; MPFC = medial prefrontal cortex; PCL = paracentral lobule; PostCG = postcentral gyrus; HES = Heschl gyrus; CAU = caudate nucleus; STG = superior temporal gyrus; SFGdor = dorsolateral part of superior frontal gyrus.

For the decreasing links (i.e., functional connectivity decreasing from the 1^st^ to the 2^nd^ half), the multivariate analysis yielded a significant Group × Time interaction effect, *F* (55, 3575) = 1.43, *p* = 0.02, for the AUDI/LANG, and an expected, significant time effect, *F* (55, 3575) = 2.16, *p* < 0.0001. Post-hoc analysis of the Group × Time interaction for the AUDI/LANG showed that the group effect was significant for the 2^nd^ half of the time series data (*p* = 0.04), but was not significant for the 1^st^ half of the data (*p* = 0.28). While the overall group effect across all dependent variables was not significant for the SEN and VIMG, partially due to a number of potentially less-relevant connections (i.e., not associated with mind wandering) being included in the model, the univariate analysis for the 2^nd^ half of the data yielded 11 functional links that exhibited a significant group effect for SEN and VIMG in addition to the AUDI/LANG (Fig. 5A). Four links associated with the SEN were connected between bilateral MPFC, left paracentral lobule, and right post-central gyrus. Four links associated with the AUDI/LANG were connected between bilateral MPFC, right Heschl gyrus, left superior temporal gyrus, and bilateral caudate nucleus. For the VIMG, 3 links were connected between bilateral MPFC, right superior temporal gyrus, and left dorsolateral superior frontal gyrus. Thus, among the 11 decreasing links that were associated with particular thought domains, 8 were connected to the MPFC, consistent with previous studies indicating its extensive patterns of connectivity between MPFC and other sensory modalities^31^,^32^. These significant group effects on functional connectivity of the decreasing links for the 2^nd^ half of the time series data indicated that participants in the higher percentage group exhibited more negative functional connectivity in the majority of the connections relative to participants in the lower percentage group (Fig. 5B and C). The exception to this pattern involved two links between right Heschl gyrus and bilateral MPFC in which higher functional connectivity was observed among participants who reported higher percentage of AUDI/LANG thought content. Additional statistical results of the univariate analysis are presented in Supplementary Table S2. For the increasing links (i.e., functional connectivity increasing from the 1^st^ to the 2^nd^ half), the expected time effect was significant, *F* (25, 1625) = 2.31, *p* = 0.0002. However, neither the group nor the Group × Time interaction effects were significant.

To explore whether we were able to observe comparable findings using seed-based connectivity analysis, we chose the MPFC as the seed region of interest to assess the correspondence between voxel-wise fMRI signals and mind wandering of different domains. Methods and results of the seed-based analysis are shown in the Supplementary Information (in the section of “Seed-based analysis”). Overall, the seed-based connectivity analysis qualitatively reproduces the major findings from the matrix-based analysis.

Altogether, the results obtained from the multivariate multiple regression analysis suggest that 1) the functional connectivity of 11 links (the majority of which connected to the MPFC) was associated with sustaining the spontaneous thoughts in particular domains: AUDI/LANG, SEN, and VIMG, 2) participants who reported spending more time in the AUDI/LANG, SEN, or VIMG exhibited a more negative functional connectivity associated with several links, compared to participants who reported spending less time in each of those same thought domains, and 3) when we examined links for which the functional connectivity tended to decrease over the course of the scan, the relationship between reported thought content and functional connectivity varied between earlier and later portions of the resting-state time series data, with relationships more pronounced in the second half. It is worth noting that, without relying on any a priori hypothesis or pre-selected seeds, we were able to identify the important roles of MPFC for mind wandering, largely in agreement with previous studies^15^,^16^,^22^. We believe that our analytical approach is complementary to the methods used in previous studies, and our proposed discovery-driven matrix-based connectivity analysis is a powerful tool that can potentially add new knowledge to resting-state fMRI and mind wandering research.

## Discussion

### Negative functional connectivity and sustaining of spontaneous thoughts

What do these results suggest for the potential underlying mechanism responsible for sustaining spontaneous thoughts during resting-state fMRI? Overall, participants who reported spending more time in spontaneous thought domains tended to exhibit more negative functional connectivity in the majority of links compared to participants who reported spending less time in each of those same thought domains (Figs 3B,C and 5B). Furthermore, a number of connections from the decreasing links (i.e., functional connectivity decreasing from the 1^st^ to the 2^nd^ half) were related to the continuity of spontaneous thoughts (Fig. 5). The results suggest that this decrease in functional connectivity might be a potential mechanism underlying the maintenance of particular types of spontaneous thoughts during resting-state fMRI, and this mechanism appears to be comparable across different thought domains.

The observed negative functional connectivity associated with the maintenance of mind wandering might be explained by a recently proposed decoupling hypothesis, which postulates that our attention is decoupled or shifted from processing events in the external world to self-generated mental activity to ensure the continuity of mind wandering^33^,^34^,^35^,^36^. Previous support for the decoupling hypothesis primarily comes from electroencephalography (EEG) studies during cognitive tasks^37^,^38^,^39^. In these EEG studies, task-related attention during cognitive activity was characterized by amplitude of event-related potentials (ERPs)^37^,^38^ and phase-locking consistency across task trials^39^. The amplitude of ERPs was reduced for participants who engaged in greater amounts of task-unrelated thought^37^,^38^, and task-unrelated thought was associated with a reduction in the trial-to-trial phase-locking consistency to visual events^39^. The findings of these studies illustrate that mind wandering during cognitive tasks is accompanied by a decrease in the processing of task-related information.

Taken together with previous studies^37^,^38^,^39^,^40^, the negative connectivity between brain regions observed in the current study can be interpreted as evidence for increased functional segregation between cortical systems subserving opposite goals or competing representations^41^. The functional segregation might reflect reciprocal modulation or inhibition/suppression through direct or indirect anatomical connections^42^,^43^,^44^. It is to be noted that, while negative correlations have been associated with data preprocessing methods using global signal regression^45^,^46^, a number of studies^42^,^47^,^48^,^49^,^50^, including this current study, observed negative correlations even in the absence of global signal regression. Therefore, the negative connectivity observed in this study could not be an artifact introduced by a global signal regression procedure. Collectively, the evidence presented above has provided initial support for the associations between negative functional connectivity and the underlying neural processes for sustaining mind wandering during resting state fMRI scans.

### Neural correlates of sustaining spontaneous thoughts during resting-state fMRI

In this study, we identified two sets of functional connections that were related to the continuity of spontaneous thoughts during resting-state fMRI. First, functional connections between the left insula and bilateral caudate nuclei, associated with AUDI/LANG, were identified from the whole-brain, whole resting-state fMRI time series data (Fig. 3A). Second, 11 connections with the majority of links connected to the MPFC, along with a number of domain-specific regions, were identified among the links which exhibited decreased connectivity in the 2^nd^ half of the time series data (Fig. 5A). The connectivity of these links during the second half of the scan was related to sustaining of spontaneous thoughts in the AUDI/LANG, SEN, or VIMG domains.

Previous studies have shown that the insula is highly interconnected with the striatum, including the caudate nucleus^51^,^52^. Both the insula and striatum are complex structures and have been implicated in a wide range of autonomic, affective, sensorimotor, self-referential, and cognitive processes, including language-related function^53^,^54^,^55^. In an extensive review of previous neuroimaging data, Price^56^ described a set of brain regions that participate in regulating language-related function. Among the language-related regions, the left insula is specifically involved in articulatory planning, whereas the bilateral caudate nuclei are associated with suppression of unintended responses^56^. In addition to their involvement with language-related function, there is recent evidence indicating that the striatum plays a critical role in brain state maintenance, whereas the insula has a major role in switching between states^30^. In relation to our study, we found negative connectivity between bilateral caudate nuclei and the left insula in participants who reported spending more time in AUDI/LANG and positive connectivity among the same links in participants who reported spending less time in this spontaneous thought (Fig. 3B and C). It is possible that, to support the continuity of mind wandering state, the caudate nuclei might exert suppressive effects on the insula to prevent from switching between brain states. Future studies are warranted to elucidate the dynamics between these two brain regions involved maintaining a state of mind wandering.

Among the additional 11 functional links that were related to greater continuity of specific domains of spontaneous thoughts, 8 of them were connected to the MPFC. The MPFC is a hub within the default mode network^3^ and has been implicated in self-related processing, such as the retrieval of remote and recent memory associated with autobiography or other self-referential processes, judgments about self and others, and simulations of social interaction^21^,^57^,^58^,^59^,^60^,^61^. Furthermore, the MPFC is characterized by a high-level interconnectivity with multiple sensory modalities such as primary/secondary auditory, somatosensory, and visual cortices^31^,^32^. This multi-modal convergence observed in the MPFC provides an anatomical ground to support the role of the MPFC together with other domain-specific brain regions in the maintenance and representation of spontaneous thoughts across different domains. Therefore, together with previous studies^10^,^15^,^16^,^22^, we propose that the MPFC is a nodal point that serves to support different domains of spontaneous thoughts during resting state.

### Mind wandering and variance in resting-state functional connectivity

While we were able to identify functional links related to participant-reported mind wandering behavior from the whole fMRI time series data by assuming temporal uniformity of functional connectivity, we found significant temporal differences in a number of links (Fig. 4). Crucially, some of the decreasing links that were equivalently associated with multiple domains of spontaneous thoughts could only be identified from the 2^nd^ half of the time series data. This indicates that differences in functional connectivity for the identified links not only manifest between individuals with different levels of self-reported spontaneous thought across specific domains, but also manifest throughout the fMRI time course profile within an individual. In the current study, the fMRI time points were divided into two halves, which revealed an overall trend of decreased functional connectivity for the 11 domain-specific links from the 1^st^ to the 2^nd^ half. Future research might include recently developed fMRI protocols of higher temporal sampling and advanced analytic procedures^62^ aimed at deconstructing time points into more non-overlapping segments, which may lead to more accurate assessment of dynamic changes in functional connectivity due to mind wandering.

As an exploratory analysis, we divided our dataset 2 (i.e., the one with the longest resting-state fMRI scan time of 520 sec: Supplementary Table S3) into four 130-sec segments for segment-specific connectivity measurement. Across the 11 domain-specific functional links of dataset 2, connectivity of the 1^st^ quarter of time series data was significantly more positive (or less negative) compared to the other 3 quarters (*p* < 0.0001), and the connectivity was not significantly different among the 2^nd^, 3^rd^, and 4^th^ quarters (Supplementary Fig. S1), suggesting that dynamic changes of functional connectivity in these links likely occurred between the 1^st^ and 2^nd^ quarters (i.e., centered around 130 sec after fMRI scans began). This observation could also explain why the maintenance of mind wandering was most pronounced in the 2^nd^ half of the scan because the 2^nd^ half of time series data for all 3 datasets began after 130 sec.

Overall, our findings associated with temporal differences in connectivity are consistent with the concept that non-static functional connectivity existed in a number of connections^23^,^24^,^25^,^27^. Further, our findings suggest that the temporal variations in functional connectivity are to some degree of neuronal origin and could be linked with the continuity of mind wandering. In addition, previous studies have found considerable between-subject variability in resting-state connectivity among healthy participants^63^,^64^. Our results suggest this between-subject variance in functional connectivity might be partially attributable to variations in spontaneous thought domain and the time spent in a specific spontaneous thought.

### Limitations and Future Directions

Our findings provide a preliminary framework for characterizing the maintenance and cerebral representation of mind wandering during resting-state fMRI. These findings, although insightful and interesting, require replication in larger samples. Several limitations should be acknowledged while interpreting our results. First, the assessment of mind wandering did not account for when the reported mind wandering occurred in the course of the fMRI scan. Future studies that combine real-time resting-state fMRI and probe measures of mind wandering during fMRI scans will be needed to measure the association between transitions of functional connectivity (e.g., from more positive to more negative correlations) and the onset of mind wandering. Second, the mind wandering behavior was determined by self report after the fMRI scan; thus, it could potentially be subject to recall bias. Third, our current study focused on associations between functional connectivity and sustaining of mind wandering. Future investigations using non-invasive brain stimulation techniques (e.g., transcranial magnetic stimulation) to modulate brain activities involved in sustaining of mind wandering are warranted to establish causal relationships between functional connectivity and mind wandering^65^,^66^.

## Conclusion

This study demonstrates that sustained mind wandering during resting-state fMRI is associated with decoupled or negative functional connectivity involving the caudate nucleus, insula, MPFC and other domain-specific brain regions. Our findings provide insights into mind wandering at the large-scale network level, and highlight the importance of including the estimated percentage of time and domain of spontaneous thoughts during resting-state fMRI into functional connectivity analyses. Future studies will need to account for these thought processes in analyses of fMRI signals during group comparisons and correlations between functional connectivity and behavioral measures. We expect that the power to detect group differences and to identify imaging-based biomarkers, especially for individual participants and clinical populations, can be enhanced by addressing the variability of mind wandering during resting-state fMRI.

## Methods

### Participants

The study’s participants included 72 right-handed healthy adults (mean age 55 ± 18 years, 37 males) from three unpublished datasets. Participants were excluded if they had a history of neurological or psychiatric disorder; unstable or untreated medical illness (including uncontrolled diabetes or treatment-resistant hypertension); any contraindication to MRI, such as claustrophobia or metallic implants; or raw score less than 27 on the Mini-Mental State Exam^67^. The institutional review board at Duke University Medical Center approved the study, and all data collection and analyses were carried out in accordance with the approved study protocol and the guidelines of the Helsinki Declaration. All of the participants provided written informed consent prior to participation.

### Resting-state questionnaire

Each participant completed a post-resting-state-fMRI interview using the adapted ReSQ^13^ to assess the content of spontaneous thoughts during the resting-state-fMRI scan. Participants were asked to estimate the proportion of time (on a 0–100% scale) spent during the resting-state-fMRI scan in each of the following five mental activities: AUDI/LANG, VIMG, SEN, MUS, and NUM. AUDI/LANG is defined as remembering/imagining words, sentences or conversations spoken by oneself or others, or talking to oneself with one’s own voice without overt production. VIMG refers to having thoughts in the form of visual images. These visual images could be associated with memory, ongoing learning, or planning of upcoming events. SEN is related to attention attracted by somatosensory sensations such as sensory information from the face or body, or position and movement of our body parts. MUS and NUM is defined as thinking related to music and numbers, respectively. For each participant, the data were log-transformed to normalize population variance. The questionnaires were completed within 30–60 minutes after the resting-state-fMRI scan.

### Imaging protocols

Resting-state-fMRI was performed on a 3 Tesla GE scanner. Each participant was scanned on a single occasion, lying still with eyes fixated on a centrally located crosshair. Participants were scanned using three echo planar imaging (EPI) protocols summarized in Supplementary Table S3. The potential influence of confounders (i.e., dataset and age) was controlled in the following analysis.

### fMRI data analysis

The preprocessing of fMRI data was conducted through the Duke Brain Imaging and Analysis Center preprocessing pipelines based on the tools from the Oxford Centre for Functional MRI of the Brain’s Software Library (FSL version 5.0.1, www.fmrib.ox.ac.uk/fsl) and locally developed Matlab codes (Mathworks, Natick, MA, USA). The first 4 volumes were discarded in order to reach the T1 steady state. The data were corrected for slice-timing differences and motion (six parameters: three translations and three rotations), and were registered to the Montreal Neurological Institute (MNI) 152 template using a 12 degrees of freedom affine transformation implemented in FSL’s Linear Image Registration Tool. All subsequent analyses were conducted in the MNI standard space. We regressed out the 6-parameter rigid body head motion (obtained from motion correction), the averaged time course profiles in the white matter, and the averaged time course profiles in the cerebrospinal fluid regions to reduce non-neuronal contributions to BOLD correlations^68^. We also removed constant offsets and linear drift. Time domain signals with their frequencies less than 0.08 Hz were retained.

A schematic diagram of our matrix-based functional connectivity analysis procedures is illustrated in Fig. 2. For each participant, the preprocessed low-frequency fMRI data were parceled into a set of 90 brain regions using the AAL template^28^. Each participant’s BOLD time series was averaged within each brain region. We used Pearson correlation as the metric of association between the time series for each pair of the 90 brain regions. This resulted in a 90 × 90 correlation matrix with 4005 ([90 × 89]/2) unique inter-regional correlation coefficients (*r*). These inter-regional *r* values were transformed to the normal distribution by Fisher’s z transform for further statistical inference^69^. Because age varied widely across participants, we regressed out age-related signals of functional connectivity and then used the residuals for the following statistical analysis.

### Statistical analyses

The first goal of the study was to identify functional connections that exhibited a significant group effect (i.e., higher percentage group vs. lower percentage group) on functional connectivity derived from the whole resting-state fMRI time series data (see Part I in Fig. 2). For each individual connection, we performed a multiple linear regression analysis to examine the group effects on functional connectivity. Each regression model included 5 independent factors (i.e., group effects of each thought domain), 1 covariate (i.e., dataset), and 1 dependent variable (i.e., functional connectivity value). The independent factor was coded as −1 (lower percentage group) and 1 (higher percentage group). The regression model was performed 4005 times, and multiple comparisons were corrected by an FDR of 0.05^29^.

The second goal of the study was to investigate whether there was an interaction effect between group and timing of the resting-state fMRI time series (see Part II in Fig. 2). First, we divided each participant’s time series data into halves (i.e., the 1^st^ half and the 2^nd^ half of the time series data). For each half, as for the whole time series data, the preprocessed fMRI data were parceled using the AAL template^28^, and 4005 ([90 × 89]/2) correlation coefficients were estimated for each half of the time series data of each participant. We then examined differences in functional connectivity between the 1^st^ half and the 2^nd^ half of the time series data by performing paired sample *t* tests on each connectivity value of the 4005 inter-regional functional links. The significance criterion was set at a *p*-value of 0.05 applying Bonferroni correction for multiple comparisons (i.e., alpha = 0.05/4005 ≈ 0.000012), minimizing false positives. The resultant networks were converted into 2 binary matrices (increasing and decreasing links) and used as inclusive masks in the subsequent analysis. Within each resultant set of links, the Group × Time interaction effects on functional connectivity were examined using multivariate multiple regression analysis. The multivariate multiple regression estimated a single regression model with more than one dependent variable (in a single step, where false positives associated with repeated assessments in conventional univariate multiple regression could be inherently eliminated). This approach allowed us to take into account the relationships among several dependent variables and conduct tests of the coefficients across different variables. The regression model included 5 independent factors (i.e., group effects of each thought domain), 1 repeated factor (i.e., time: 1^st^ half vs. 2^nd^ half of the time series data), 1 covariate (i.e., dataset), and functional connectivity values of a set of links (decreasing or increasing links) as dependent variables. The multivariate multiple regression analysis yielded two outputs: 1) results of multivariate analysis of variance that tested the overall group effects on functional connectivity across all dependent variables; and 2) results of univariate analysis that examined the group effect on functional connectivity of each individual dependent variable for each thought domain.

## Additional Information

**How to cite this article:** Chou, Y.-h. *et al*. Maintenance and Representation of Mind Wandering during Resting-State fMRI. *Sci. Rep.* **7**, 40722; doi: 10.1038/srep40722 (2017).

**Publisher's note:** Springer Nature remains neutral with regard to jurisdictional claims in published maps and institutional affiliations.

## Supplementary Material

Supplementary Information
