# Supplementary material for: Maintenance and Representation of Mind Wandering during Resting-State fMRI
Source: Sci Rep. 2017 Jan 12;7:40722. doi: 10.1038/srep40722 (PMC5227708; doi:10.1038/srep40722)

Supplementary Information

Maintenance and Representation of Mind Wandering during Resting-State fMRI

Ying-hui Chou, Mark Sundman, Heather E. Whitson, Pooja Gaur, Mei-Lan Chu,
Carol P. Weingarten, David J. Madden, Lihong Wang, Imke Kirste, Marc Joliot,
Michele T. Diaz, Yi-Ju Li, Allen W. Song, and Nan-kuei Chen

Supplementary Table S1.

Brain connections that exhibited a significant difference in functional connectivity between the 1st and the 2nd halves of the fMRI time series data

Functional links	1st half	2nd half
Decreasing Links		
**R-MPFC ↔ L-PCL**	**0.05 ± 0.06**[a]	**-0.22 ± 0.05**
**L-MPFC ↔ L-PCL**	**0.05 ± 0.05**	**-0.24 ± 0.05**
**R-MPFC ↔ R-PostCG**	**0.07 ± 0.06**	**-0.23 ± 0.05**
**L-MPFC ↔ R-PostCG**	**0.11 ± 0.06**	**-0.16 ± 0.05**
**R-MPFC ↔ R-STG**	**0.22 ± 0.07**	**-0.08 ± 0.04**
**L-MPFC ↔ R-STG**	**0.27 ± 0.07**	**-0.02 ± 0.05**
**R-MPFC ↔ R-HES**	**0.14 ± 0.06**	**-0.12 ± 0.03**
**L-MPFC ↔ R-HES**	**0.21 ± 0.06**	**-0.05 ± 0.04**
**R-HES ↔ L-CAU**	**0.16 ± 0.07**	**-0.07 ± 0.05**
**L-STG ↔ R-CAU**	**0.07 ± 0.06**	**-0.16 ± 0.04**
**L-SFGdor ↔ R-STG**	**0.16 ± 0.08**	**-0.12 ± 0.06**
R-MPFC ↔ L-PreCG	0.21 ± 0.06	-0.04 ± 0.04
L-MPFC ↔ L-PreCG	0.24 ± 0.06	-0.05 ± 0.04
R-MPFC ↔ L-PostCG	0.08 ± 0.05	-0.16 ± 0.04
L-MPFC ↔ L-PostCG	0.01 ± 0.05	-0.26 ± 0.04
R-MPFC ↔ R-CUN	-0.15 ± 0.06	-0.39 ± 0.05
L-SFGdor ↔ L-PreCG	0.48 ± 0.06	0.19 ± 0.05
L-SFGdor ↔ L-PostCG	0.11 ± 0.05	-0.17 ± 0.05
R-HES ↔ R-CAU	0.07 ± 0.06	-0.20 ± 0.04
L-HES ↔ R-CAU	-0.01 ± 0.05	-0.23 ± 0.04
R-TPOsup ↔ R-MOG	-0.08 ± 0.05	-0.35 ± 0.05
R-TPOsup ↔ R-SOG	-0.10 ± 0.05	-0.36 ± 0.04
R-TPOsup ↔ R-CAL	-0.10 ± 0.05	-0.30 ± 0.05
L-TPOsup ↔ R-MOG	0.02 ± 0.05	-0.16 ± 0.04

Supplementary Table S1 continues

Supplementary Table S1 continued

L-CAU ↔ L-PreCG	0.15 ± 0.06	-0.10 ± 0.04
L-MTG ↔ L-GPA	0.11 ± 0.05	-0.13 ± 0.04
R-ANG ↔ R-PreCG	0.29 ± 0.06	0.06 ± 0.05
R-ANG ↔ L-PCL	0.21 ± 0.05	-0.01 ± 0.03
Increasing Links		
R-ROL ↔ L-FFG	-0.31 ± 0.05	-0.12 ± 0.04
R-PreCG ↔ L-FFG	-0.32 ± 0.06	-0.10 ± 0.04
L-LING ↔ R-CUN	0.40 ± 0.05	0.60 ± 0.04
R-PreCG ↔ L-IOG	-0.26 ± 0.07	-0.01 ± 0.05
L-HES ↔ L-IOG	-0.12 ± 0.04	0.15 ± 0.05
L-STG ↔ L-IOG	-0.13 ± 0.05	0.19 ± 0.04
L-ORBsupmed ↔ L-PUT	0.04 ± 0.05	0.24 ± 0.05
L-TPOsup ↔ L-ORBinf	0.48 ± 0.06	0.70 ± 0.06
L-REC ↔ R-SMA	-0.33 ± 0.05	-0.10 ± 0.05
L-TPOsup ↔ L-ITG	-0.17 ± 0.06	0.11 ± 0.04

Note. Functional connections that exhibited a significant difference in connectivity between the higher and the lower percentage groups are in bold. L = left; R = right; MPFC = medial prefrontal cortex; PCL = paracentral lobule; PostCG = postcentral gyrus; STG = superior temporal gyrus; HES = Heschl gyrus; CAU = caudate nucleus; PreCG = precentral gyrus; CUN = Cuneus; SFGdor = dorsolateral part of superior frontal gyrus; TPOsup = temporal pole of superior temporal gyrus; MOG = middle occipital gyrus; SOG = superior occipital gyrus; CAL = calcarine sulcus; MTG = middle temporal gyrus; GPA = globus pallidus; ANG = angular gyrus; ROL = Rolandic operculum; FFG = fusiform gyrus; LING = lingual gyrus; IOG = inferior occipital gyrus; ORBsupmed = medial orbital part of superior frontal gyrus; PUT = putamen; ORBinf = orbital part of inferior frontal gyrus; REC = gyrus rectus; SMA = supplementary motor area.
[a]Standard error.

Supplementary Table S2.

Univariate results of multivariate multiple regression analysis for the 2nd half of the time series data

Functional links	Thought domain	$F(1, 65)$	p
R-MPFC ↔ L-PCL	SEN	5.51	0.02
L-MPFC ↔ L-PCL	SEN	5.28	0.02
R-MPFC ↔ R-PostCG	SEN	4.99	0.03
L-MPFC ↔ R-PostCG	SEN	4.96	0.03
L-MPFC ↔ R-HES	AUDI/LANG	4.27	0.04
R-MPFC ↔ R-HES	AUDI/LANG	4.88	0.03
L-STG ↔ R-CAU	AUDI/LANG	4.12	0.05
R-HES ↔ L-CAU	AUDI/LANG	6.37	0.01
L-MPFC ↔ R-STG	VIMG	4.81	0.03
R-MPFC ↔ R-STG	VIMG	5.22	0.03
L-SFGdor ↔ R-STG	VIMG	4.25	0.04

Note. L = left; R = right; MPFC = medial prefrontal cortex; PCL = paracentral lobule; PostCG = postcentral gyrus; HES = Heschl gyrus; CAU = caudate nucleus; STG = superior temporal gyrus; SFGdor = dorsolateral part of superior frontal gyrus; SEN = somatosensory awareness; AUDI/LANG = auditory mental imagery/inner language; VIMG = visual mental imagery.

Supplementary Table S3.

Resting-state fMRI scanning parameters

	Dataset 1 (*n* = 18)	Dataset 2 (*n* = 25)	Dataset 3 (*n* = 29)
Sequence	T2*-weighted parallel EPI with an acceleration factor of 2	T2*-weighted parallel EPI with an acceleration factor of 2	T2*-weighted EPI
TR (sec)	4	5	2
TE (msec)	35	25	25
In-plane matrix size	160 x 160	128 x 128	64 x 64
Slice number	56	78	36
Voxel size (mm^3)	1.5 x 1.5 x 3	1.8 x 1.8 x 1.8	4 x 4 x 4
Number of time points	70	104	182
Acquisition time (sec)	280	520	364

Note. EPI = echo planar imaging; TR = repetition time; TE = echo time.

Supplementary Figure S1: Mean functional connectivity of each quarter of the resting-state fMRI data across 25 participants from dataset 2. Abbreviations: L = left; R = right; MPFC = medial prefrontal cortex; PCL = paracentral lobule; PostCG = postcentral gyrus; HES = Heschl gyrus; CAU = caudate nucleus; STG = superior temporal gyrus; SFGdor = dorsolateral part of superior frontal gyrus.

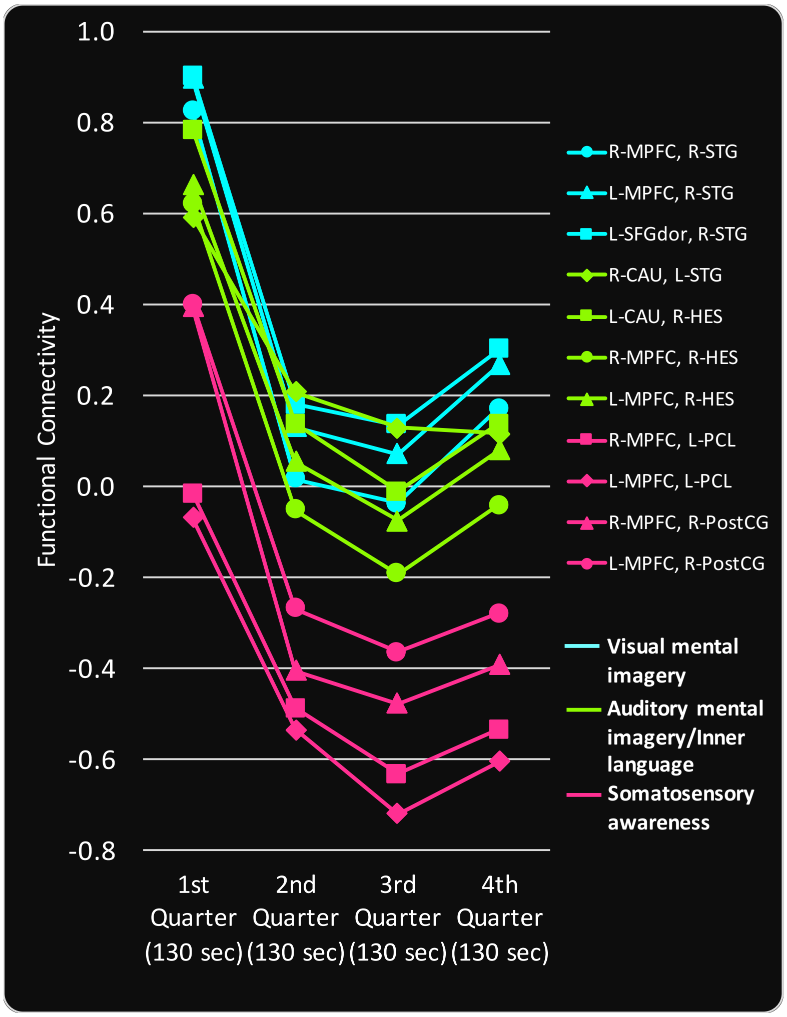

Seed-based analysis

The purpose of this supplementary analysis was to explore whether conventional seed-based analysis would be able to identify connectivity patterns that were comparable to the results produced by matrix-based connectivity analysis (see the Results section). The medial prefrontal cortex (MPFC) was chosen as the seed region of interest to analyze the the second-half of the time series data for all the voxels within 90 AAL regions, so that the results could be compared with the matrix-based connectivity analysis. The methodology and corresponding results are described below.

Image data analysis

We carried out seed-based analysis using FEAT (FMRI Expert Analysis Tool) Version 5.0.9, part of FSL (FMRIB's Software Library, www.fmrib.ox.ac.uk/fsl). The MPFC seed region of interest was created in standard space based on the AAL template (AAL IDs 23 and 24). For each participant, the preprocessed time courses extracted from the voxels in the seed region were used as an explanatory variable in a first-level analysis. For the higher-level analysis, we performed a multiple regression analysis using the general linear model as implemented in FEAT. We modeled the following contrasts: 1) AUDI/LANG — lower percentage group > higher percentage group; 2) VIMG — lower percentage group > higher percentage group; and 3) SEN — lower percentage group > higher percentage group. Age and dataset were included as covariates in the group-level analysis.

Results

We identified several brain regions that exhibited a significantly lower connectivity to the medial prefrontal cortex in the higher percentage group compared with the lower percentage group. These regions included the left language and auditory areas (central/parietal opercular cortex) for the AUDI/LANG, and the right temporal occipital cortex for the VIMG, as shown in the Supplementary Figure S2A. Multiple comparisons were corrected with cluster significance of $p < 0.05$, $Z > 2.3$. When a more liberal threshold was used (uncorrected, $p < 0.01$), we identified the right precentral and postcentral gyrus for the SEN (Supplementary Figure S2B). The findings are qualitatively similar to the findings from the matrix-based connectivity analysis.

It is worth noting that we identified the occipital cortex across the three domains of spontaneous thoughts (AUDI/LANG, VIMG, and SEN). In other words, participants in the higher percentage group

exhibited a significantly lower functional connectivity between the occipital cortex and the MPFC. Although participants were instructed to fixate their eyes on the crosshair during the resting-state fMRI scan, it was possible that participants might not fixate their eyes on the crosshair during mind wandering. Future studies (e.g., with eye tracking) are needed to better understand the roles of occipital cortex in mind wandering.

Table S4.

Clusters showing brain regions that exhibited a significantly lower connectivity to the medial prefrontal cortex in the higher percentage group compared with the lower percentage group (corrected cluster significance of $p < 0.05$, $Z > 2.3$)

Domain	Cluster size	*p*	Cluster center of gravity	Regions
AUDI/LANG	1458	0.0000004	-54, -28, 25	Left central/parietal opercular cortex
AUDI/LANG	384	0.03	-17, -81, 18	Lateral occipital cortex
VIMG	572	0.003	31, -57, -6	Right temporal occipital cortex
SEN	1134	0.000008	-32, -68, -8	Left temporal occipital cortex

Supplementary Figure S2. Brain regions that exhibited a significantly lower connectivity to the medial prefrontal cortex in the higher percentage group compared with the lower percentage group.

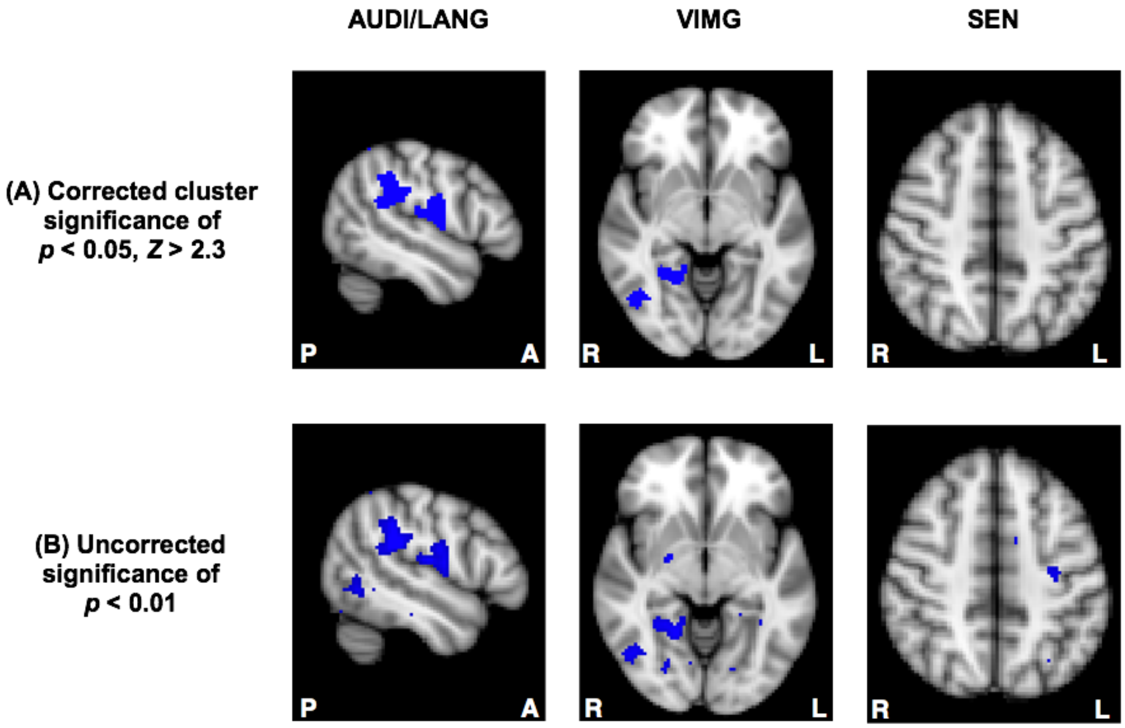

Supplement: Supplementary Information [file srep40722-s1.pdf]
